# Ethanol Infusion in the Vein of Marshall for Persistent Atrial Fibrillation Ablation: Evidence From Randomized Controlled Trials

**DOI:** 10.1111/jce.70090

**Published:** 2025-09-03

**Authors:** Carolina Saleiro, Sérgio Barra, Bárbara Oliveiros, Patrícia Alves, João Ferreira, Natália António, Luís Elvas, Lino Gonçalves, Miguel Valderrábano, Pedro A. Sousa

**Affiliations:** ^1^ Pacing & Electrophysiology Unit, Cardiology Department Coimbra's Hospital and University Center Coimbra Portugal; ^2^ Cardiology Department Hospital da Luz Arrábida Vila Nova de Gaia Portugal; ^3^ ICBR, Faculty of Medicine University of Coimbra Coimbra Portugal; ^4^ Houston Methodist DeBakey Heart and Vascular Center and Research Institute Houston Texas USA

**Keywords:** atrial fibrillation, catheter ablation, ethanol infusion, persistent, vein of Marshall

## Abstract

**Background:**

Pulmonary vein isolation (PVI) is the cornerstone of catheter ablation (CA) for atrial fibrillation (AF), yet outcomes remain suboptimal in persistent AF patients. Ethanol infusion in the vein of Marshal (VoM), an embryological remnant implicated in AF pathogenesis, may enhance ablation efficacy.

**Objective:**

To evaluate the effectiveness of VoM ethanol infusion in patients with persistent AF.

**Methods:**

We conducted a systematic review and meta‐analysis of randomized controlled trials (RCT) comparing CA with versus without VoM ethanol infusion in patients undergoing first‐time ablation of persistent AF. The primary endpoint was freedom from any atrial arrhythmia in a 12‐month follow‐up.

**Results:**

Four RCTs including 1045 patients were analyzed (VoM + CA—535 patients vs. CA alone—510 patients). Ethanol infusion in the VoM significantly increased freedom from atrial arrhythmias (RR 1.21; 95% CI 1.010–1.32; *p* < 0.0001, NNT 10) and reduced the need of a repeat procedure (RR 0.63; 95% CI 0.45–0.87; *p* = 0.005). Mitral isthmus (MI) block was more frequently achieved in the VoM group (RR 1.30; 95% CI 1.03–1.65; *p* = 0.03) There was no significant difference in the rate of major complications (2.8% vs. 3.5%, RR 0.72; 95% CI 0.37–1.43; *p* = 0.35, NNH 138), although overall complications were more frequent in the VoM ethanol infusion group (RR 2.25; 95% CI 1.08–4.70; *p* = 0.03).

**Conclusion:**

When added to CA, ethanol infusion in the VoM improves freedom from arrhythmia without increasing the risk of major complications. These findings may support its integration into ablation strategies for persistent AF.

**Abbreviations**
AF: atrial fibrillation
CA: catheter ablation
CI: confidence interval
EIVOM: ethanol infusion in the vein of Marshal
MI: mitral isthmus
PROMPT‐AF: Prospective Randomized Comparison Between Upgraded 2C3L Versus PVI Approach for Catheter Ablation of Persistent Atrial Fibrillation
PVI: pulmonary vein isolation
RCTs: randomized controlled trials
RR: risk ratio
VENUS: Vein of Marshall Ethanol for Untreated Persistent AF
VoM: vein of Marshal

## 1 Background

Atrial fibrillation (AF) is the most common sustained cardiac arrhythmia, associating with significant morbidity and mortality [1]. Catheter ablation (CA) is the treatment of choice in symptomatic AF patients who are refractory to antiarrhythmic drug therapy, with pulmonary vein isolation (PVI) being the cornerstone of AF ablation [1, 2]. However, despite improvements in AF ablation in the last years, freedom from atrial arrhythmia remains low, particularly in patients with persistent and long‐standing persistent AF [3].

Despite the lack of robust evidence supporting the routine use of additional ablation beyond PVI [4, 5, 6], a recent survey showed that two‐thirds of physicians perform additional lesions beyond PVI in de novo patients submitted to persistent AF ablation, with more than 90% performing additional lesions in redo procedure [7].

The Vein of Marshall (VoM), an embryological remnant of the left superior vena cava, has been associated as a potential source of AF triggers and is linked to autonomic nerve fibers involved in the pathogenesis and maintenance of AF [8, 9]. The Vein of Marshall Ethanol for Untreated Persistent AF (VENUS) trial showed the benefit of adding ethanol infusion in VoM (EIVOM) to CA. Nevertheless, as the ablation protocol used in the trial was extensive and nonstandardized, assessing the role of VoM ethanol infusion was difficult [10].

Although several meta‐analyses on EIVOM have been published in recent years, they had significant limitations. These meta‐analyses included heterogeneous populations with both paroxysmal and persistent AF patients and were mostly based on observational and cohort data while including only a single randomized controlled trial (RCT) [11, 12, 13, 14, 15]. Furthermore, some focused on different endpoints, such as achieving perimitral block [12]. These limitations likely contribute to the ongoing uncertainty surrounding the role of EIVOM in persistent AF ablation, as noted in the most recent AF Expert Consensus Statement on Catheter and Surgical Ablation of AF [2].

However, new RCTs evaluating EIVOM were recently published, offering potential new evidence [16, 17, 18]. This updated systematic review with meta‐analysis of RCTs aimed to assess the added value of adjunctive EIVOM in persistent AF ablation.

## 2 Methods

### 2.1 Search Strategy and Selection

This meta‐analysis was conducted in accordance with the PRISMA statement. The prospective meta‐analysis protocol was registered in the International Prospective Register of Systematic Reviews (PROSPERO CRD420251040299). Ethics approval was not required as no individual patient data were collected.

A systematic literature search was performed in May 2025 across Medline, Embase, and the Cochrane Library databases using the keywords “atrial fibrillation” and “ablation” and “vein of Marshall” or “ethanol ablation.” No restrictions were applied regarding language, publication date, or publication status. The search was limited to RCTs, clinical trials and studies. Additionally, we manually reviewed the reference list of relevant studies to identify other potentially eligible studies.

### 2.2 Study Design

Studies were considered for inclusion in the analysis if they met the following criteria: (i) RCT design; (ii) enrollment of patients with persistent AF undergoing a first ablation procedure; (iii) comparison between AF CA with adjunctive EIVOM versus CA only; and (iv) reported data on atrial tachyarrhythmia recurrence with a minimum follow‐up of 12 months.

Exclusion criteria were (i) non‐RCTs; (ii) RCTs including patients with both persistent and paroxysmal AF or atrial flutter; (iii) studies including redo ablation procedures; and (iv) studies published only as abstracts or as oral conference presentations. Two authors (P.S. and N.A.) independently screened all retrieved publications to determine eligibility. Any disagreement regarding study eligibility was resolved through discussion, with input from a third author (P.A.S.).

### 2.3 Data Extraction

The data extracted from the final studies included the first author's last name, number of patients in the EIVOM and CA groups, inclusion date, ablation strategy, primary endpoint, follow‐up duration, and complications. Data on baseline characteristics—including age, gender, CHA_2_DS_2_‐VASC score, AF duration, left atrial diameter, left ventricular ejection fraction, procedural details (procedure and fluoroscopy times), and complications—were considered relevant for cohort characterization and were collected when available.

### 2.4 Quality Assessment

The risk of bias for the included articles was independently assessed by two authors (P.S. and N.A.), following the Cochrane Collaboration's “Risk of bias 2” tool. Any disagreements were resolved by a third author (P.A.S.). The following domains were evaluated: random sequence generation, allocation concealment, blinding of participants and personnel, blinding of outcome assessment, incomplete outcome data, and selective reporting biases. Each domain was classified as having a “low,” “high,” or “unclear” risk of bias. The detailed quality assessment for each study is presented in the “risk of bias summary” (Figure S1).

### 2.5 Outcomes

The primary endpoint was freedom from any sustained atrial arrhythmia during a 12‐month follow‐up, defined as a composite of AF, atrial tachycardia, or flutter.

Secondary endpoints included: (i) need of redo procedure; (ii) achievement of mitral isthmus (MI) block; and (iii) safety outcomes. Subanalysis was performed to evaluate arrhythmia recurrence by type, reporting freedom from AF and freedom from atrial tachycardia or flutter as separate outcomes. The safety endpoint included both major complications and the overall number of complications. Major complications were defined as pericardial tamponade or effusion requiring interventional treatment, any thromboembolic event, gastroesophageal fistula, symptomatic pulmonary vein stenosis, persistent phrenic nerve palsy, or vascular events requiring interventional treatment or resulting in prolonged hospitalization. The total number of complications included major complications plus pericarditis/pericardial effusion not requiring intervention and minor vascular events. We also assessed total procedure and fluoroscopy times.

### 2.6 Statistical Analysis

Continuous variables are expressed as mean ± standard deviation for normally distributed data or median and interquartile range for non‐normally distributed data, and categorical variables are expressed as frequencies or percentages. Pooled risk ratios (RR) and 95% confidence intervals (CI) were estimated based on a random effects meta‐analysis and were obtained from the pooled adjusted RR of primary studies. Weighted mean differences were used to pool continuous endpoints. Statistical significance was accepted for *p* values < 0.05. The *I* ^2^ statistic was used to assess statistical heterogeneity across studies (moderate heterogeneity was considered present for values between 25% and 50% and high heterogeneity above 50%). Meta‐analysis was conducted according to the random‐effects model. For meta‐regression, each eligible predictor was assessed individually through simple meta‐regression, given the limited number of studies included in the analysis. We used Review Manager 5.4 (Nordic Cochrane Centre, London, UK) software to perform statistical analyses. Meta‐regression was performed using IBM SPSS, Version 29.

## 3 Results

### 3.1 Study Selection and Quality Assessment

**Figure 1:**
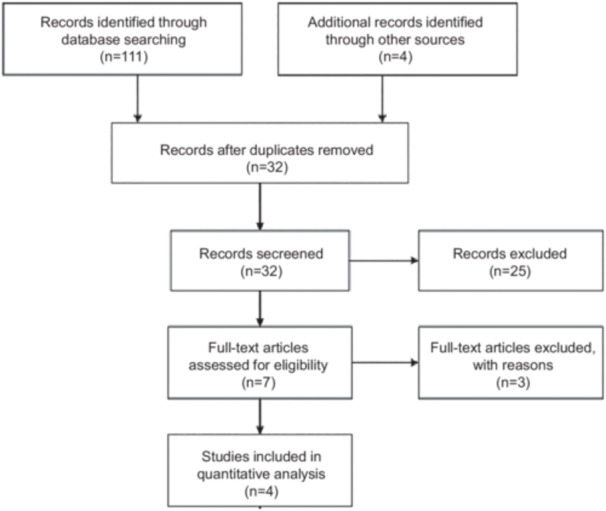
Study selection flowchart.

A total of 111 studies were identified: after removal of duplicates, a total of 32 studies were considered. Of these, 25 were excluded after title/abstract screening for not meeting the inclusion criteria. Three additional studies were excluded after full‐text review: two were nonrandomized trials and one did not report follow‐up data. The study selection flowchart is shown in Figure 1. Four RCTs were included in the analysis [10, 16, 17, 18]. Overall, all studies were considered as having a low risk of bias (Figure S1).

### 3.2 Study Characteristics

The study design and characteristics of the RCTs included in the analysis are detailed in Table 1. A total of 1045 patients with persistent AF were included, of whom 535 patients underwent EIVOM plus CA while 510 patients had CA alone. The selected studies were published between 2020 and 2025. In two of the studies (*n* = 308), the CA group received PVI alone [17, 18], whereas the other two included additional ablation lines [10, 16]. The baseline characteristics are listed in Table 2. No significant differences between groups were observed. The feasibility of EIVOM ranged from 83.7% to 97% [10, 16, 17, 18].

**Table 1:** Study design and characteristics.

Trial, year, country	Groups, *N*	Inclusion date	Ablation strategy	Primary endpoint	Follow‐up	Adverse events
Valderrábano et al. [10] VENUS Multicenter United States of America	CA + EIVOM = 185 vs. CA = 158	October 2013–June 2018	RF + ethanol—Additional ablation lesions beyond those required to achieve PVI were delivered in 95.7% of the patients. Successfully EIVOM in 83.7% of the patients. vs. RF—Additional ablation lesions beyond those required to achieve PVI were delivered in 95.6% of the patients.	Freedom from any atrial tachycardia was 49.2% in the CA combined with EIVOM group compared with 38% in the CA group (*p* = 0.04).	12 months 12‐lead ECG at 1, 3, 6, 9, and 12 months. Continuous 1‐month monitoring at 6 and 12 months.	Intraprocedural: 11 vascular access complications and 3 pericardial effusion. 7 patients had cerebrovascular events. Subacute pericardial effusion requiring pericardiocentesis occurred in 4 patients. Symptomatic pericarditis not requiring drainage occurred in 11 patients in the EIVOM and in 6 in the CA group.
Zuo et al. [16] Single center China	CA + EIVOM = 45 vs. CA = 44	—	RF + ethanol—same protocol as control group plus EIVOM. Successfully EIVOM in 91% of the patients. vs. RF—Antral PVI plus a roof line, posterior MI line, intracoronary sinus, and CTI ablation.	Secondary endpoint: at 1 year, 17.8% patients who underwent EIVOM had recurrent atrial tachycardia, compared with 31.8%; in the CA (*p* < 0.01).	12 months 12‐lead ECG and 24‐h holter electrocardiogram monitoring at 3, 6, and 12 months.	No serious adverse events. Pericardial effusion without need for interventional treatment similar between groups (2 of 45 vs. 1 of 44; *p* > 0.05).
Sang et al. [17] PROMPT‐AF Multicenter China	CA + EIVOM = 246 vs. PVI only = 249	August 2021–July 2023	RF + ethanol—PVI plus linear ablation of roof, MI, and CTI + EIVOM. EIVOM was successfully performed in 85% of the patients. vs. RF—PVI only	Īn total, 70.7% of the patients in the EIVOM and 61.5% of the patients in CA remained free from atrial arrhythmias (*p* = 0.045).	12 months Single‐lead ECG patch for a period of at least 24 h each week during the 12 months.	No difference in procedural‐related adverse events between groups (*p* = 0.15). Seven patients in the EIVOM group experienced pericarditis/pericardial effusion compared with none in the PVI group.
Derval et al. [18] The Marshal‐Plan Single center France	CA + EIVOM = 59 vs. PVI only = 59	January 2020–November 2022	RF + ethanol—PVI plus linear ablation of dome, MI, and CTI + EIVOM. EIVOM was successfully performed in 97% of the patients. vs. RF—PVI only.	Freedom from recurrence of atrial arrhythmia occurred in 86.4% of the patients in the EIVOM group compared to 66.1% of the patients assigned to PV isolation only (*p* = 0.012).	12 months Follow‐up visits at 3, 6, 9, and 12 months with a 12‐lead ECG. 30 s ECG recording transmission every week by a trans telephonic monitor. Recording and transmission of any symptomatic arrhythmia.	Major procedure‐related adverse events did not differ between groups (1.7% vs*.* 1.7%; *p* = 1.0). In the CA group, one patient developed an oesophagopericardial fistula. In the EIVOM, one patient had severe groin hematoma requiring radioembolization and blood transfusion. Most complications were pericarditis with no significant differences between arms.

Abbreviations: CA, catheter ablation; CTI, cavotricuspid isthmus; ECG, electrocardiogram; EIVOM, ethanol injection in the vein of Marshal; FA, radiofrequency; MI, mitral isthmus; PROMPT‐AF, Prospective Randomized Comparison Between Upgraded 2C3L Versus PVI Approach for Catheter Ablation of Persistent Atrial Fibrillation; PVI, pulmonary vein isolation; VENUS, Vein of Marshall Ethanol for Untreated Persistent AF trial.

**Table 2:** Baseline population characteristics.

References	Patients, *N*	Age, years	Male, *N* (%)	CHA_2_DS_2_‐VASC score	AF duration, months	LVEF, %	LAD, mm
Valderrábano et al. [10]	CA + EIVOM = 185	66.6 ± 9.6	137 (74)	2.9 ± 1.6	—	52.1 ± 10.1	44.8. ± 7.9
	CA = 158	66.4 ± 9.9	124 (78)	2.6 ± 1.6	—	53.4 ± 9.4	47.0 ± 7.5
Zuo et al. [16]	CA + EIVOM = 45	63.0 ± 6.3	25 (55.6)	1.8 ± 1.1	26 ± 14	57.0 ± 5.2	42.3 ± 3.1
	CA = 44	62.8 ± 6.2	26 (59.1)	1.8 ± 0.9	28 ± 16	58.1 ± 5.1	43.3 ± 3.1
Sang et al. [17]	CA + EIVOM = 246	61.3 ± 9.9	180 (73.2)	1 (1‐2)	12 (5‐24)	61.3 ± 7.6	42.8 ± 6.1
	CA = 249	61.0 ± 9.5	181 (72.7)	1 (0‐2)	12 (4‐24)	61.1 ± 8.0	42.8 ± 4.5
Derval et al. [18]	CA + EIVOM = 59	66 ± 8	48 (80)	2 ± 1	10 ± 18	51 ± 12	—
	CA = 59	65 ± 8	51 (85)	2 ± 1	7 ± 6	56 ± 10	—

Abbreviations: AF, atrial fibrillation; CA, catheter ablation; EIVOM, ethanol injection in the vein of Marshal; LAD, left atrium diameter; LVEF, left ventricular ejection fraction.

### 3.3 Effect of VoM Ethanol Ablation on Freedom From Any Atrial Arrhythmia

**Figure 2:**
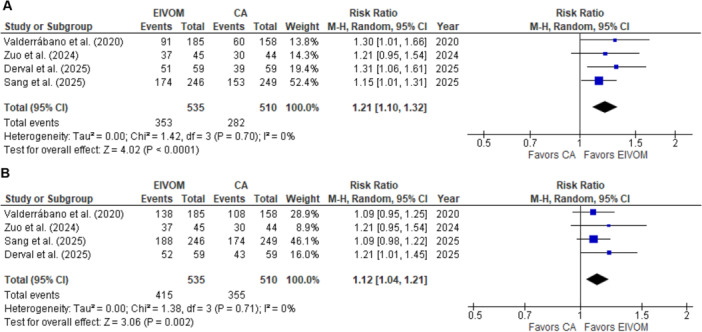
Pooled analysis for freedom from (A) any atrial tachycardia and (B) atrial fibrillation comparing EIVOM plus CA and CA alone. Numbers displayed represent RRs with 95% CIs. EIVOM, ethanol injection in the vein of Marshal; CA, catheter ablation; RR, risk ratio; CI, confidence intervals. Please note that for easy of understanding, EIVOM is represented “left” and CA is presented “right” in the forest plot.

All studies reported freedom from any sustained atrial arrhythmia, after a blanking period of 3 months and during a 1‐year follow‐up. Therefore, a total of 1045 patients were evaluated for this endpoint. EIVOM plus CA was associated with a significantly higher freedom from any atrial arrhythmia, without heterogeneity, (RR 1.21; 95% CI 1.01–1.32; *I* ^2^ = 0%; *p* < 0.0001, NNT 10) (Figure 2A) compared to CA alone.

**Figure 3:**
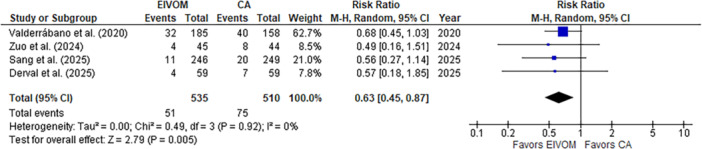
Pooled analysis for repeat procedures comparing EIVOM plus CA and CA alone. Numbers displayed represent RRs with 95% CIs. EIVOM, ethanol injection in the vein of Marshal; CA, catheter ablation; RR, risk ratio; CI, confidence intervals.

We compared the impact of EIVOM on freedom from AF and the need of a repeat procedure. Freedom from AF recurrence was significantly higher in the EIVOM plus CA compared to CA alone (RR 1.12; 95% CI 1.04–1.21; *I* ^2^ = 0%; *p* = 0.002) (Figure 2B). Freedom from atrial tachycardia or flutter was similar between groups (Figure S2). All studies reported data on redo procedures. Notably, EIVOM combined with CA was associated with a significantly lower need of redo procedures compared to CA alone (RR 0.63; 95% CI 0.45–0.87; *I* ^2^ = 0%; *p* = 0.005) (Figure 3). There was no heterogeneity in the analysis. Freedom from any atrial arrythmia and need of redo procedures were independent of age, gender, AF duration, left atrial diameter, and left ventricular ejection fraction (Table S1).

### 3.4 Successful Mitral Isthmus Block

**Figure 4:**
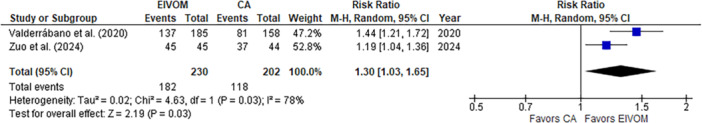
Pooled analysis for mitral line block at the end of the procedure comparing EIVOM plus CA and CA alone. Numbers displayed represent RRs with 95% CIs. EIVOM, ethanol injection in the vein of Marshal; CA, catheter ablation; RR, risk ratio; CI, confidence intervals. Please note that for easy understanding, EIVOM is represented “left” and catheter ablation is presented “right” in the forest plot.

Two studies reported comparative data on MI block at the end of the procedure, with a total of 432 patients evaluated for this endpoint [10, 16]. Overall, bidirectional MI block was more frequently achieved in the CA plus EIVOM group (79.1% vs. 58.4%) (RR 1.30; 95% CI 1.03–1.65; *I* ^2^ = 78%; *p* = 0.03), although with high heterogeneity (Figure 4).

### 3.5 Safety Endpoint—Periprocedural Complications

**Figure 5:**
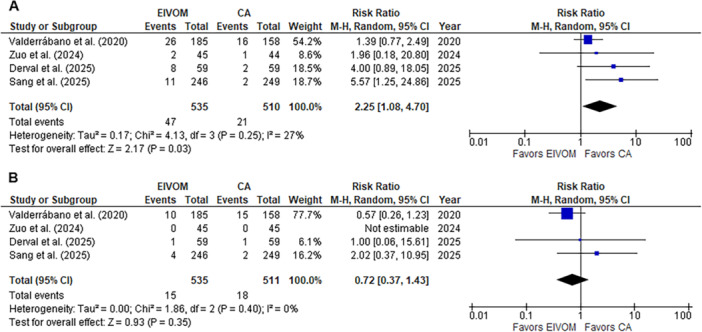
Pooled analysis for (A) overall and (B) major comparing EIVOM plus CA and CA alone. Numbers displayed represent RRs with 95% CIs. EIVOM, ethanol injection in the vein of Marshal; CA, catheter ablation; RR, risk ratio; CI, confidence intervals.

All studies reported data on periprocedural complications. The overall complication incidence was significantly higher in the EIVOM plus CA group compared to CA alone (8.8% vs. 4.1%; RR 2.25; 95% CI 1.08–4.70; *I* ^2^ = 27%; *p* = 0.03) (Figure 5A), which was mainly driven by an increase in pericardial effusion/pericarditis not requiring drainage in the EIVOM plus CA group (4.9% vs. 1.7%; RR 2.53; 95% CI 1.02–6.29; *I* ^2^ = 11%; *p* = 0.05) (Figure S3). Nevertheless, there was no significant difference between groups in the incidence of major complications, without heterogeneity (2.8% vs. 3.5%, RR 0.72; 95% CI 0.37–1.43; *I* ^2^ = 0%; *p* = 0.35, NNH 138) (Figure 5B).

### 3.6 Fluoroscopy Time and Procedure Duration

Data on fluoroscopy time and procedure duration were reported for 956 and 1045 patients, respectively. As expected, fluoroscopy time was significantly longer in the EIVOM plus CA group (mean difference 10.39 min; 95% CI 8.69–12.08; *I* ^2^ = 0%; *p* < 0.00001) (Figure S4A). Procedure duration was longer in the EIVOM plus CA group, although differences were not statistically significant, with very high heterogeneity (mean difference 21.89 min; 95% CI 13.95–56.83; *I* ^2^ = 97%; *p* = 0.22) (Figure S4B).

## 4 Discussion

In this systematic review and meta‐analysis of RCTs comprising over 1000 patients with persistent AF, the main findings were: (i) EIVOM in addition to CA increases 1‐year freedom from any sustained atrial arrhythmia; (ii) VoM ethanol ablation increases the likelihood of achieving MI bidirectional and reduces the need of redo procedures; (iii) VoM ethanol ablation may associate with increased overall complication rate, without however increasing the risk of major complications.

AF is a global pandemic with its prevalence expected to double over the next 50 years [19]. It is associated with significant morbidity and mortality, presenting a considerable burden on patients, public health systems, and healthcare economies. PVI alone is an effective treatment for paroxysmal AF ablation, with single‐procedure success rates of up to 90% at 1 year when optimized workflows are used [20, 21]. However, achieving comparable outcomes in patients with persistent AF remains a significant challenge. Several factors may explain the disappointing results observed in RCTs assessing different strategies in persistent AF patients: (i) the disease is heterogeneous, with varying degrees of atrial remodeling; (ii) non‐PV triggers and perpetuators likely play a role and explains why it is so difficult to find a “one fits all” ablation strategy; (iii) difficulty to obtain durable bidirectional block across ablated lines, particularly at the MI [4, 5, 10, 16]. However, despite the lack of robust evidence supporting the routine use of additional ablation to PVI, the perception that PVI alone may be insufficient to obtain high freedom from atrial arrhythmias in persistent AF patients or that specific tailored ablation strategies may be required for selected patients may explain why ablation beyond PVI continues to be performed by many European electrophysiologists [5]. Therefore, there is a need to find new approaches with proven efficacy in reducing arrhythmia recurrence in these patients.

The VoM obliquely connects the posterolateral wall of the left atrium to the proximal coronary sinus, harboring autonomic nerve fibers, myocardial bundles, blood vessels, fibrous tissue, and ganglion cells, all of which play a significant role in the pathogenesis and maintenance of AF [8, 22, 23]. Ethanol ablation of VoM has been shown to eliminate AF triggers and regional autonomic innervation, as well as to facilitate the achievement of durable MI bidirectional block [9, 24, 25]. The VENUS trial was the first to demonstrate the benefit of adding EIVOM to CA. VENUS trial results may have been confounded by the use of extensive, nonstandardized ablation at the operator's discretion—including linear lines, complex fractionated atrial electrogram ablation, and posterior wall isolation—which were allowed in both the control and then EIVOM groups. Non‐PVI ablation lesions were not significantly different between the groups, so EIVOM was the main differentiating factor. Since the VENUS trial was designed to test the value of EIVOM when added to an aggressive lesion set, it is possible that non‐PVI lesions might have impacted the results [10]. More recently, three additional RCTs evaluating VoM ethanol infusion in persistent AF patients have been published, further substantiating the added value of this approach in reducing AF recurrence [16, 17, 18]. In two of these trials—PROMPT‐AF (Prospective Randomized Comparison Between Upgraded 2C3L Versus PVI Approach for Catheter Ablation of Persistent Atrial Fibrillation) and The Marshall Plan—VoM ethanol infusion was incorporated as part of an anatomical ablation strategy which included ablation lines, while the control group underwent PVI alone [17, 18]. Notably, both studies reported a significant increase in freedom from atrial arrhythmia during a 1‐year follow‐up in the EIVOM groups compared to the control groups (71% and 86% vs*.* 61% and 66%, respectively) [17, 18]. Prior trials had shown no significant benefit of additional lesion sets when added to PVI in persistent AF ablation [4, 5, 6]. Therefore, it is possible that in PROMPT‐AF and Marshall Plan trials the observed differences in outcomes were primarily driven by the addition of EIVOM, although the contribution of additional ablation lines cannot be excluded. With the current technology, the likelihood of achieving durable conduction block along ablation lines is markedly higher, as observed in these two recent trials, in which acute bidirectional block exceeded 87% for all lines [17, 18].

Our meta‐analysis corroborates these findings, suggesting that the addition of ethanol ablation in the VoM increases freedom from atrial arrhythmia at 1 year. Notably, this approach also significantly reduced the likelihood of requiring repeat procedures, which has important implications in reducing waiting lists and decreasing healthcare costs in public health systems. This represents a meaningful step forward in the treatment of patients with persistent AF. Pulsed field ablation may also offer similar benefits in reducing costs and waiting times; however, the ability to effectively ablate epicardial structures, along with the safety profile—particularly for the MI—and the long‐term durability of lesions remain to be established [26, 27]. Further RCTs are warranted to directly compare these two strategies.

Perimitral flutter is likely the most common reentrant tachycardia following AF ablation [28, 29]. However, achieving durable bidirectional MI block remains challenging due to different factors, such as tissue thickness, anatomic variability, and the presence of the epicardial Marshall bundle [30, 31]. These difficulties contribute to suboptimal success rates and hamper the success rates of persistent AF ablation, as incomplete lesion sets can be proarrhythmic. In a subanalysis of VENUS trial, achieving perimitral block with EIVOM was associated with a remarkably increased difference between randomization groups, in favor of the EIVOM group [25]. Our meta‐analysis suggests that EIVOM associates with an increased probability of achieving MI block supporting its routine inclusion when treating patients with persistent AF or perimitral flutter.

The overall risk of adverse events was significantly higher in VoM ethanol infusion group. However, major complications did not differ between groups and were in line with results from other studies [32, 33]. The increase in overall complications appears to be primarily driven by a higher incidence of pericarditis and pericardial effusion not requiring drainage in the VoM group, likely reflecting an inflammatory response due to ethanol extravasation into the pericardial space or due to the additional linear ablations performed in this cohort. These findings may support the consideration of prophylactic anti‐inflammatory therapy (e.g., with colchicine) [34], and routine postprocedure echocardiography monitoring to mitigate inflammation‐related complications. As with many technically demanding procedures, feasibility and outcomes improve with operator experience. A subanalysis of the VENUS trial demonstrated higher rates of freedom from atrial arrhythmias in high‐volume centers performing VoM ablations [25].

This systematic review and meta‐analysis provides clinically meaningful data. Unlike previous meta‐analyses which were limited by the inclusion of observational studies and heterogenous patient populations, our study only included RCTs enrolling patients with persistent AF. As a result, our analysis demonstrated low heterogeneity and offers a more robust assessment of the EIVOM added value in persistent AF ablation. Our findings support the addition of EIVOM to CA as an effective strategy to improve outcomes in this challenging population, as well as to reduce the need of repeat procedures and improve the chance of achieving durable MI block. Although the rate of major complications did not differ between groups, the overall increase in procedural complications underscores the need for adequate training, center expertise, and the consideration of prophylactic measures to minimize risks. Taken together, the balance leans in favor of adding EIVOM for persistent AF ablation. As this technique becomes more widely adopted, it has the potential to significantly improve outcomes in persistent AF ablation.

### 4.1 Limitations

This meta‐analysis has several limitations that should be acknowledged. First, although only RCTs were included, there was a degree of heterogeneity in the ablation protocols across studies, with ablation in control groups ranging from PVI‐only to more extensive linear ablations. Nevertheless, results remained consistent regardless of the ablation strategy in the control group (Figure S5). Second, one of the included studies was primarily designed to evaluate MI block and could be underpowered to assess freedom from atrial arrhythmia as a clinical endpoint [16]. Third, strategies for arrhythmia monitoring during follow‐up differed among studies, which could introduce additional bias. However, since monitoring protocols were applied equally to both treatment and control groups within each study, this limitation is unlikely to have affected our main findings. Finally, the follow‐up duration was limited to 12 months, and thus long‐term efficacy and durability of the benefits associated with VoM ethanol infusion remain unclear. Future RCTs with standardized protocols and extended follow‐up are required to address these gaps.

## 5 Conclusion

In patients with persistent AF, the addition of EIVOM to standard CA significantly improves freedom from atrial arrhythmia recurrence, while reducing the need for repeat procedures and increasing the chance of achieving MI block. Although this approach associates with a higher risk of overall complication, major complications are not increased.

## Conflicts of Interest

S.B. has received training grants from Biosense Webster and Biotronik. J.F. has received consulting fees from Medtronic, Biotronik, and Microport. N.A. has received consulting fees from Microport and Abbott. M.V. is supported by the Charles Burnett III, DeBakey Funds, and Lois and Carl Davis Centennial Chair endowments, NIH R61‐HL164873 and R01‐HL168277. M.V. has received consulting fees and research funding from Biosense Webster. P.A.S. has received consulting fees from Abbott, Biosense Webster, Boston Scientific, and Medtronic. All other authors have reported that they have no relationships relevant to the contents of this paper to disclose.

## Supporting information


**Table S1 Meta‐regression**. **Figure S1** – Risk of bias summary. All studies were considered to have low risk of bias. **Figure S1** – Risk of bias summary. All studies were considered to have low risk of bias. **Figure S3** ‐ Pooled analysis for pericarditis and pericardial effusion not requiring drainage comparing EIVOM plus CA and CA alone. **Figure S4** ‐ Pooled analysis for (A) total procedural time and (B) fluoroscopy time comparing EIVOM plus catheter ablation and catheter ablation alone. **Figure S5** Pooled analysis for freedom from any atrial tachycardia comparing EIVOM plus CA and (A) control group of CA consisting of pulmonary vein isolation only; (B) control group of CA consisting of pulmonary vein isolation plus additional linear ablation.

## Data Availability

The data that support the findings of this study are available from the corresponding author upon reasonable request.
