# Supplementary material for: Ethanol Infusion in the Vein of Marshall for Persistent Atrial Fibrillation Ablation: Evidence From Randomized Controlled Trials
Source: J Cardiovasc Electrophysiol. 2025 Sep 3;36(11):3014–23. doi: 10.1111/jce.70090 (PMC12614149; doi:10.1111/jce.70090)
Supplement: Supplementary file 1 — Table S1 Meta‐regression. Figure S1 – Risk of bias summary. All studies were considered to have low risk of bias. Figure S1 – Risk of bias summary. All studies were considered to have low risk of bias. Figure S3 ‐ Pooled analysis for pericarditis and pericardial effusion not requiring drainage comparing EIVOM plus CA and CA alone. Figure S4 ‐ Pooled analysis for (A) total procedural time and (B) fluoroscopy time comparing EIVOM plus catheter ablation and catheter ablation alone. Figure S5 Pooled analysis for freedom from any atrial tachycardia comparing EIVOM plus CA and (A) control group of CA consisting of pulmonary vein isolation only; (B) control group of CA consisting of pulmonary vein isolation plus additional linear ablation. [file JCE-36-3014-s001.docx]

**Supplementary Material:**

**Table S1 – Meta-regression.**

|  | Age, years | Male, N (%) | AF Duration,  Months | LVEF, % | LAD, mm |
| --- | --- | --- | --- | --- | --- |
| Freedom from any atrial tachycardia | -4.9 (0.787) | 119.13 (0.806) | -0.76 (0.852) | 0.98 (0.777) | 0.59 (0.972) |
| Redo procedure | -10.86 (0.933) | -170.59 (0.946) | -2.3 (0.921) | 0.98 (0.97) | -0.83 (0.984) |
| Total complications | -24.13 (0.832) | 338.07 (0.856) | -6.16 (0.866) | 3.76 (0.833) | 0.79 (0.993) |

Outcome variables, expressed as number needed to treat (NNT) and number needed to harm (NNH), were found to be independent of differences in age, gender distribution, atrial fibrillation duration, left ventricular ejection fraction (FEVE) and left atrial diameter (LAD). These factors did not demonstrate statistical significance as predictors of the outcomes. The regression coefficients and their corresponding p-values, within brackes, are presented above, providing a detailed assessment of the relationships examined.


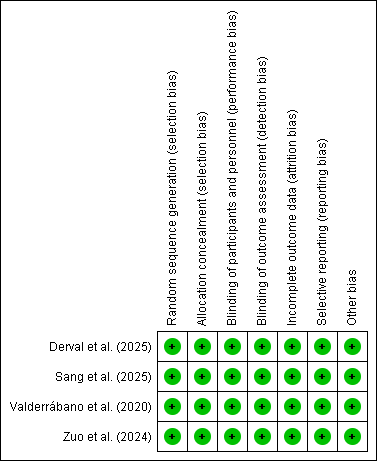


**Figure S1** – Risk of bias summary. All studies were considered to have low risk of bias.


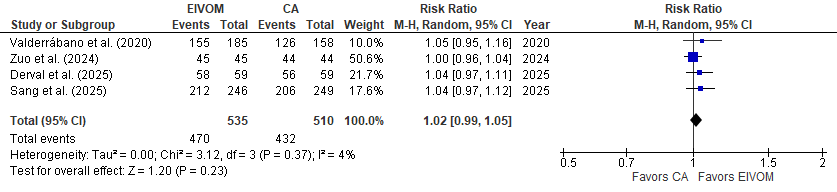


**Figure S2 -** Pooled analysis for freedom from atrial tachycardia or flutter comparing EIVOM plus CA and CA alone. Numbers displayed represent RRs with 95% CIs. EIVOM – ethanol injection in the vein of Marshal; CA – catheter ablation; RR – risk ratio; CI – confidence intervals. Please note that for easy of understanding, EIVOM is represented "left" and CA is presented "right" in the forest plot.

**
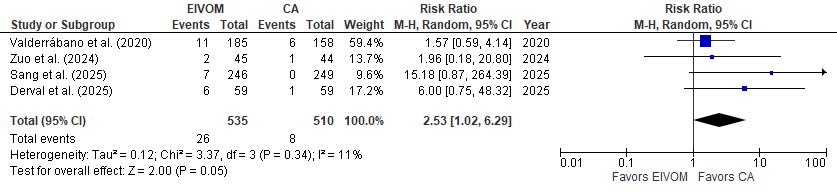
**

**Figure S3** - Pooled analysis for pericarditis and pericardial effusion not requiring drainage comparing EIVOM plus CA and CA alone. Numbers displayed represent RRs with 95% CIs. EIVOM – ethanol injection in the vein of Marshal; CA – catheter ablation; RR – risk ratio; CI – confidence intervals

**A**


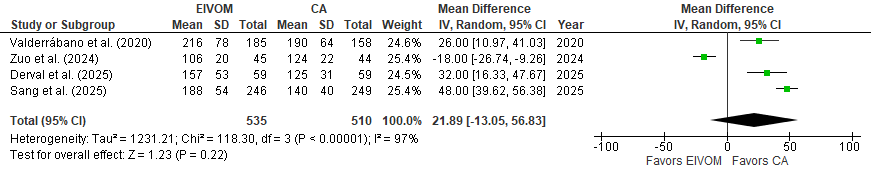


**B**

**
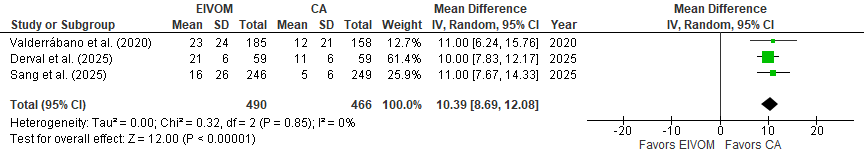
**

**Figure S4** - Pooled analysis for (A) total procedural time and (B) fluoroscopy time comparing EIVOM plus catheter ablation and catheter ablation alone. Numbers displayed represent MDs with 95% CIs. EIVOM – ethanol injection in the vein of Marshal; MD – mean differences; CI – confidence intervals

**A**


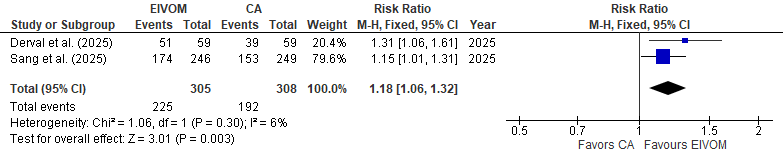


**B**


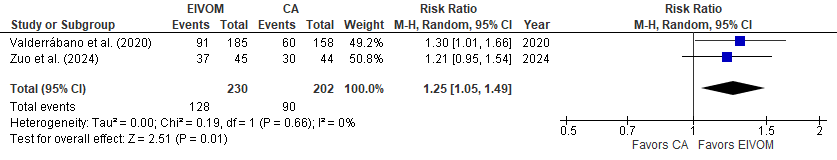


**Figure S5 -** Pooled analysis for freedom from any atrial tachycardia comparing EIVOM plus CA and (A) control group of CA consisting of pulmonary vein isolation only; (B) control group of CA consisting of pulmonary vein isolation plus additional linear ablation. Numbers displayed represent RRs with 95% CIs. EIVOM – ethanol injection in the vein of Marshal; CA – catheter ablation; RR – risk ratio; CI – confidence intervals. Please note that for easy of understanding, EIVOM is represented "left" and CA is presented "right" in the forest plot.
